# Ongoing evolution of urinary tract disorders in dromedary camels (*Camelus dromedarius*): a comprehensive illustrated sonographic overview

**DOI:** 10.3389/fvets.2025.1638275

**Published:** 2025-06-30

**Authors:** Mohamed Tharwat, Hazem M. M. Elmoghazy

**Affiliations:** ^1^Department of Clinical Sciences, College of Veterinary Medicine, Qassim University, Buraidah, Saudi Arabia; ^2^University Veterinary Hospital, Qassim University, Buraidah, Saudi Arabia; ^3^Veterinary Teaching Hospital, Faculty of Veterinary Medicine, Benha University, Benha, Egypt

**Keywords:** camels, incidence, diseases, urinary tract, ultrasonography

## Abstract

In practice, urinary tract disorders are increasingly reported in dromedary camels, particularly in regions where environmental stress and limited resources prevail. Our primary goal in writing this review was to provide a simple, accessible, and practical data tailored for veterinary clinicians working with dromedary camels in remote and resource-limited environments. These veterinarians often operate under harsh conditions such as pastoral regions of North Africa, the Middle East, and parts of South Asia, with very basic tools and limited access to advanced diagnostic equipment. Therefore, we intentionally structured this manuscript to be practical and user-friendly in such settings, emphasizing clinical relevance and practicality over exhaustive academic depth. The review is structured to highlight the main urinary disorders affecting dromedary camels, with a focus on their incidence, clinical presentation, and sonographic characteristics. While not exhaustive in academic detail, this review maintains scientific accuracy with a focus on practical application. The first section discusses the incidence and trends of various urinary tract disorders observed in dromedary camels. The second section provides an overview of the ultrasonographic anatomy of the urinary system in healthy camels. The third and main section focuses on the ultrasonographic findings associated with urinary tract disorders in affected camels that contains illustrative images of published case reports as well as clinical studies. This section is further divided into seven subsections: (1) pyelonephritis and renal abscesses, (2) urinary tract calculi, (3) urine retention, dribbling, and anuria, (4) urinary bladder rupture, (5) urethral rupture, (6) cystitis, and (7) urinary neoplasia. A thorough understanding of the pathophysiology of urinary tract diseases in dromedary camels is essential for developing effective treatment and control strategies considering normal variation or age-related differences. If appropriate, the review discusses also treatment options or preventive measures based on sonographic findings. By presenting recent research findings, this review aims to raise awareness and guide future strategies for the diagnosis and management of urinary tract diseases in dromedary camels, ultimately contributing to improved health outcomes in both domestic and wild populations.

## 1 Introduction

In dromedary camels (*Camelus dromedarius*), urinary tract diseases present a significant challenge for field veterinarians, particularly in Saudi Arabia (1). Through hospital-based affected dromedaries, previous studies by the authors have focused extensively on the sonographic diagnosis of renal diseases, as well as the normal ultrasonographic appearance of the urinary tract in this species (1, 2). Recent studies have shed light on various urinary tract disorders in camels, including pyelonephritis, hydronephrosis, urolithiasis, neoplasia, and renal abscessation (3–5). These disorders not only compromise the health and productivity of camels but also pose public health concerns, particularly due to zoonotic pathogens such as *Staphylococcus lugdunensis* and *Staphylococcus aureus* (6).

Based on the admission complains, the clinical diagnostic approach to renal disorders in camels typically involves case history, physical examination, laboratory testing, and diagnostic imaging techniques (3). However, many camels with renal disease are presented with vague histories and inconclusive laboratory findings, making diagnosis challenging (1, 2). As a result, field veterinarians often rely heavily on diagnostic imaging, particularly ultrasonography, to confirm renal conditions. However, numerous limitations affect its field utility, e.g., operator dependence and depth resolution. Sonography has proven to be a valuable non-invasive modality for the early detection of renal diseases in dromedary camels (5, 6). It enables accurate identification of several renal pathologies, such as pyelonephritis and renal abscessation, thus facilitating timely surgical intervention and treatment monitoring (3).

This review article aims to provide field veterinarians with a comprehensive, illustrated sonographic overview of urinary tract disorders in dromedary camels. We emphasize that a thorough understanding of the pathophysiology of these conditions is essential for developing effective treatment and control strategies. By presenting recent research findings, this review seeks to raise awareness, guide future diagnostic and therapeutic approaches, and ultimately contribute to improved health outcomes in dromedary camel populations.

## 2 Incidence of urinary tract diseases

On an abattoir-based investigation, a post-mortem investigation of 100 camels was conducted to assess the prevalence and characteristics of renal pathologies (7). Gross examination revealed several abnormalities, including pigmentation of the renal capsule, medullary hyperemia, subcapsular calcification, discoloration of the cortex and medulla, hemorrhage within the renal pelvis, hydatid cysts, and nephrolithiasis. Histopathological evaluation further identified lesions such as acute tubular injury, capsular melanosis, chronic interstitial nephritis, tissue calcification, hydatid cyst formation, and enhanced medullary vascularization (7). These findings suggest multifactorial etiologies, including infectious, toxic, and metabolic contributions.

In a separate study of 121 camel kidney samples, pathological changes observed included hydronephrosis, hemorrhage, vascular congestion, glomerulonephritis, hyaline degeneration, interstitial nephritis, renal cysts, tubular damage, amyloid deposits, hemosiderosis, pyelonephritis, and renal toxicity (8). Another investigation of 50 adult camels found that 33 (66%) exhibited gross kidney lesions, while 17 (34%) appeared healthy. The renal abnormalities included hemorrhage, hydronephrosis, and necrosis, with microscopic examination revealing hyalinization, glomerular shrinkage, cortical and medullary congestion, protein casts, interstitial hemorrhage, tubular cell swelling, and thickening of glomerular tufts (9). Similar, field studies reported a 72% prevalence of cystitis, with higher incidence among camels aged 3–7 years, potentially due to increased exposure to infectious agents or urinary tract anomalies (10). In Djanet province, southeastern Algeria, a study of 62 male dromedary camels—5 intact and 57 castrated—found urolithiasis in 10 castrated and 1 intact male, corresponding to a urinary calculi incidence of 17.74% (11). The higher incidence among castrated males may be linked to hormonal imbalances or dietary management, which warrants further investigation.

## 3 Ultrasound of the urinary system in healthy camels

**Figure 1:**
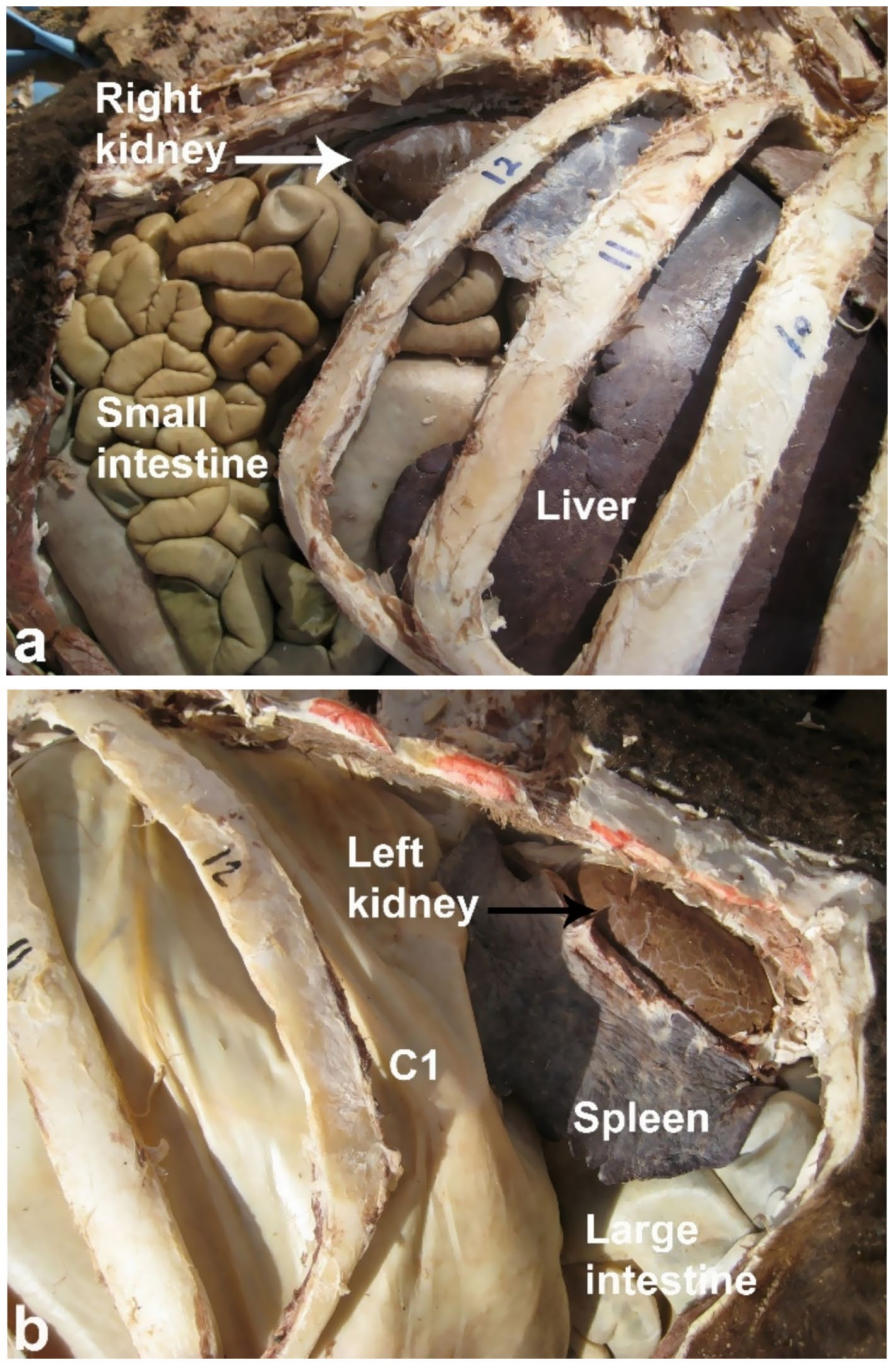
Anatomical position of the right (white arrow, **a**) and left (black arrow, **b**) kidneys in a camel carcass preserved in 10% formalin solution. C1, first gastric compartment. Adapted from (3).

**Figure 2:**
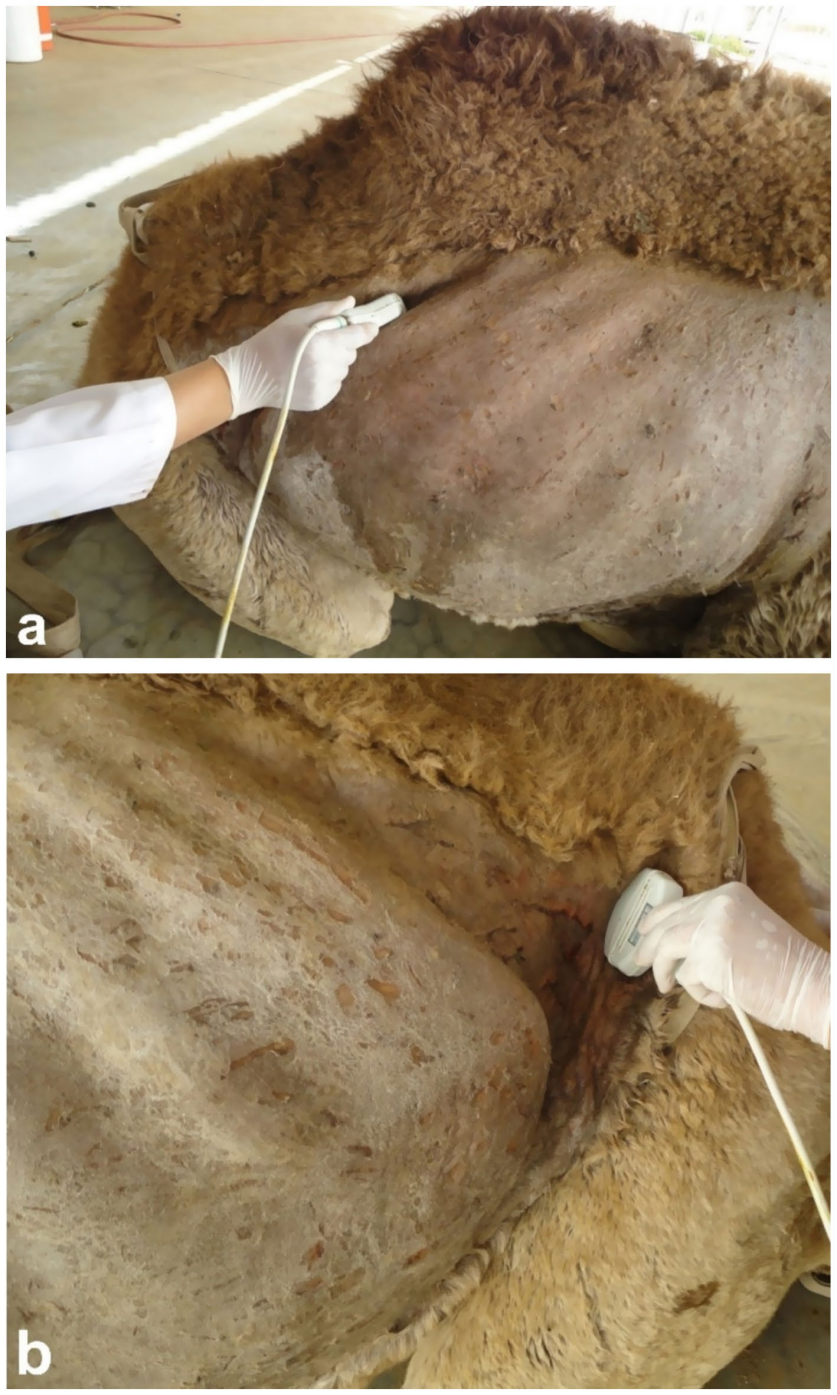
Ultrasonographic imaging of the right kidney in the upper right flank **(a)** and the left kidney in the caudal part of the left flank **(b)**. Adapted from (3).

**Figure 3:**
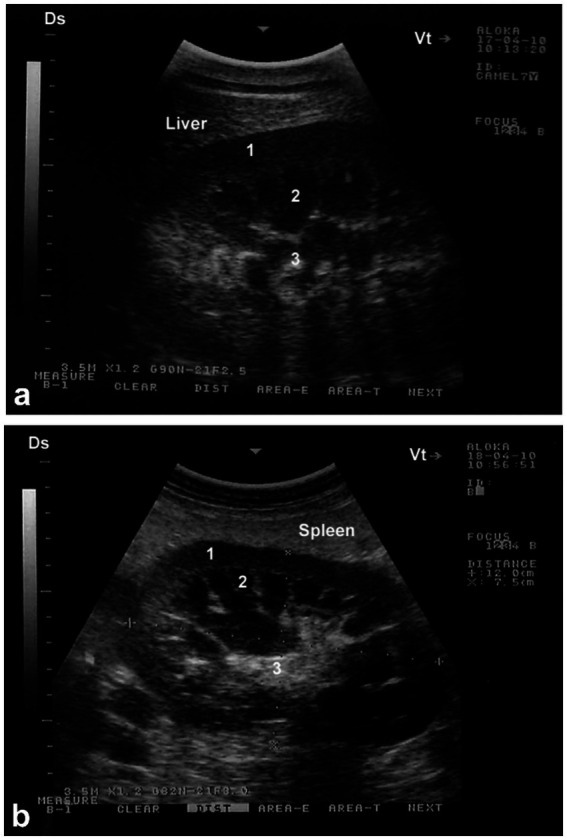
Longitudinal ultrasonograms of the right **(a)** and left **(b)** kidneys in a healthy camel. Image **(a)** was obtained from the 10th right intercostal space through the hepatic window; image **(b)** was captured from the middle of the left flank through the splenic window. 1: cortex; 2: medulla; 3: renal sinus. DS: dorsal; VT: ventral. Adapted from (3).

**Figure 4:**
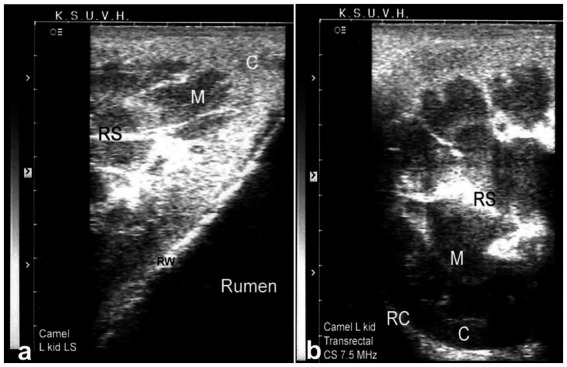
Transrectal ultrasonography of the left kidney in a healthy camel. The cranial pole of the left kidney is visible **(a)**, along with a cross-sectional view **(b)**. C: cortex; M: medulla; RS: renal sinus; RC: renal capsule; RW: rumen wall. Adapted from (3).

Sonography is a non-invasive and valuable diagnostic tool for the immediate evaluation of the urinary tract in dromedary camels. The procedure is preferred than other diagnostic methods based on the portability, safety and real-time results, but with a diagnostic limitation here such as operator dependency and difficulty in obese animals. Sonography allows detailed anatomical visualization of the kidneys, ureters, urinary bladder, penile body, and urethra. These images provide valuable information for identifying pathological conditions (6, 12). In transabdominal sonography, the right and left kidneys are typically examined through the upper right and lower left paralumbar fossae, respectively. Figure 1 shows the position of the right and left kidneys in a camel carcass preserved in a 10% formalin solution. During the examination, the transducer is held perpendicular for the right kidney and angled caudally for optimal imaging of the left kidney (Figure 2). Both kidneys can also be evaluated through the 11th intercostal space (hepatic window) and the middle left paralumbar fossa (splenic window; Figure 3). The left kidney is additionally visualized in both longitudinal and cross-sectional planes, as well as transrectally that offers high-resolution images due to proximity (Figure 4). For the transrectal examination, transmission gel is applied to the transducer, which is then covered with a rectal sleeve and inserted into the rectum. The probe is positioned ventrally, laterally, and dorsally in relation to the left kidney (3).

Because of the relatively large size of the kidneys, it is easy to differentiate clearly between the renal cortex and medulla in the vast majority of cases. The renal cortex appears hyperechoic relative to the hypoechoic medulla. The medullary pyramids have a triangular shape and are of low echogenicity than other kidney structures. In addition, the renal sinus is more echogenic, which makes it clearly distinguishable from both cortex and medulla. When compared to the neighboring hepatic and splenic parenchyma, the right and left kidneys are of lower echogenicity, respectively. When the ultrasound probe is placed on the flank in a longitudinal position, the renal hilus may be visualized; thus, facilitates the assessing of renal blood vessels or ureter origin. Through the hepatic and splenic windows, the right and left kidneys can also be scanned effectively. The left kidney may be easily accessible transrectally in most cases either in a cross section or longitudinal views (3, 6). The urinary bladder is best visualized transrectally; the wall of the bladder is seen as a smooth echoic line with normally anechoic contents. The pelvic urethra is typically difficult to visualize transrectally, except in cases of obstruction or stricture (4, 6).

## 4 Ultrasound of the urinary system in camels with urinary tract pathologies

### 4.1 Pyelonephritis and renal abscesses

In dromedary camels, pyelonephritis is a significant renal disease frequently reported in abattoir surveys, and characterized by inflammation of the kidneys and renal pelvis, typically resulting from ascending bacterial infections (3). Clinical signs include nonspecific systemic signs such as fever, lethargy, anorexia, and abnormal urine appearance such as hematuria or pyuria. Diagnosis is primarily based on ultrasonographic visualization of renal enlargement and altered parenchymal echogenicity, with antibiotic selection based on culture and sensitivity results from aspirated material. If untreated, pyelonephritis may progress to chronic renal failure. Effective management requires targeted antibiotic therapy and supportive care to prevent recurrence (3).

Renal abscesses, often secondary to bacterial infections, are also clinically significant renal complications where ultrasonography plays a vital role in early detection and therapeutic planning (6). Sonographically, abscesses appear as heterogeneous echogenic areas within the renal parenchyma, frequently featuring an anechoic center indicative of fluid or pus accumulation. The abscess margins may be irregular, and posterior acoustic enhancement is often observed. Surrounding tissue may exhibit signs of nephritis, such as cortical thickening and increased echogenicity, supporting the diagnosis. Differential diagnosis includes hematoma and neoplasia that might mimic abscess on ultrasound (3).

**Figure 5:**
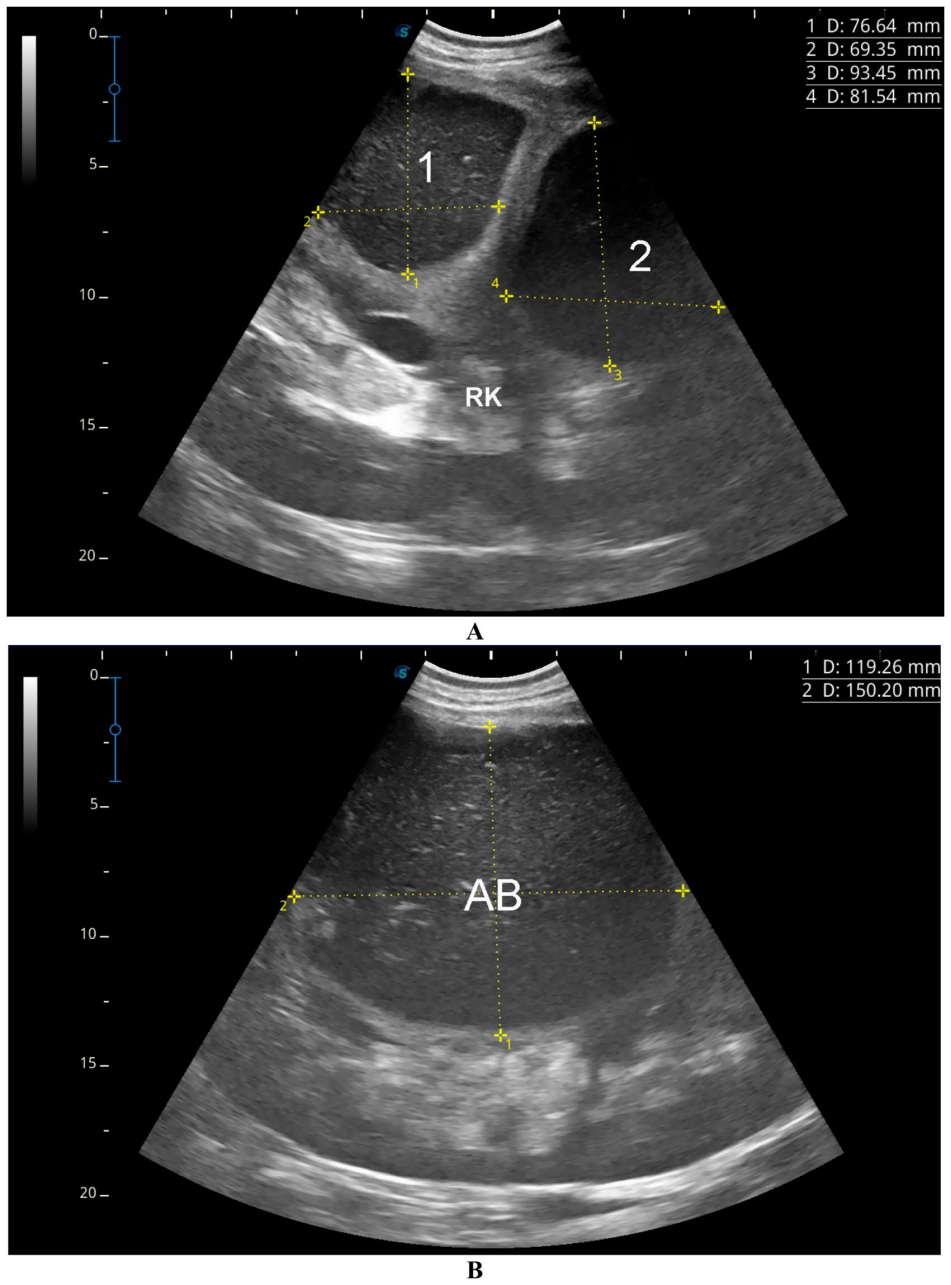
Ultrasonographic images showing renal abscesses in a camel. **(A)** Two well-defined abscesses in the right kidney, and **(B)** a large abscess in the left kidney, all containing isoechoic material. These patterns help differentiate kidney abscesses from other renal conditions and support diagnosis of pyelonephritis. Adapted from (6).

Clinically, camels with renal abscesses may present with weight loss, anorexia, constipation and emaciation. Other presentation findings may include dysuria, lameness, abdominal discomfort and skin abscesses. Laboratory findings often reveal neutrophilic leukocytosis, elevated serum proteins, blood urea nitrogen, hyperglycemia, and increased alkaline phosphatase activity. Ultrasound imaging can detect single or multiple renal abscesses compressing renal tissue and distort the renal contour, with lesions varying in echogenicity and heterogeneity (Figure 5) (6). Ultrasound-guided aspiration reliably obtains samples for bacteriological culture, which typically reveals Gram-positive cocci arranged in grape-like clusters. Biochemical analysis identifies *Staphylococcus lugdunensis* and *Staphylococcus aureus* as predominant pathogens which typically confirms the causative organism and guides appropriate antimicrobial therapy (6).

### 4.2 Urinary tract calculi

Urolithiasis is a common subclinical condition in male ruminants, especially those fed grain-rich diets or grazing on specific pastures, with prevalence rates reaching 40–60% in some populations (4). In males, urinary calculi—a multifactorial condition and a leading cause of mortality across species (13)—typically form in the kidney calyces or more frequently in the urinary bladder. In dromedary camels, small uroliths may migrate into the ureter or urethra, causing partial or complete urinary obstruction, often at the external urethral orifice or sigmoid flexure of the penis (14). The incidence and clinical impact of urolithiasis vary among species, with camels and other ruminants being particularly susceptible due to unique anatomical features that complicate diagnosis and treatment (15–20). Urinary calculi formation is commonly linked to nutritional imbalances, leading to mineral precipitation in the urinary tract (17). The narrow and tortuous urethra of camels and ruminants often impedes stone passage, resulting in obstruction, pain, impaired renal function, and potential mortality if untreated (21–25). Conventional treatments are challenged by the complex urethral anatomy in these species (26, 27). Clinical signs include urinary retention, repeated posturing, recumbency with limb paddling, perineal pulsation, preputial sheath contraction, and anuria (28).

Silica uroliths are the predominant type reported in male camels, whereas apatite and struvite stones are more common in other ruminants (17, 29, 30). Camelids most frequently experience urethral obstruction at the urethral reflection near the ischium or at the narrowed penile urethra entrance to the glans, differing from cattle that often obstruct at the sigmoid flexure and possess a urethral process absent in camelids (31–35). Khaki et al. (36) reported a low incidence (0.71%) of urinary stones in 140 camel bladders examined postmortem near Tehran. While urolithiasis is common in livestock, reported cases in camels remain rare. Both sexes can develop calculi, but obstruction is more common in males due to their narrower urethra, with stones typically lodging in the distal sigmoid flexure. Calculus composition varies geographically, with silicate, phosphate, and calcium carbonate crystals identified. Excessive silica intake was directly linked to urolithiasis in two castrated male camels (17). In a study of 62 male dromedaries, 11 had uroliths—10 castrated and 1 intact—with six stones analyzed showing smooth or rough cream-colored surfaces consistent with calcite (calcium carbonate) composition (11). Contributing factors include high concentrate diets, imbalanced calcium-phosphorus ratios, silica-or oxalate-rich pastures, vitamin A deficiency, vitamin D toxicity, reduced water intake, and elevated salt content in drinking water (4).

**Figure 6:**
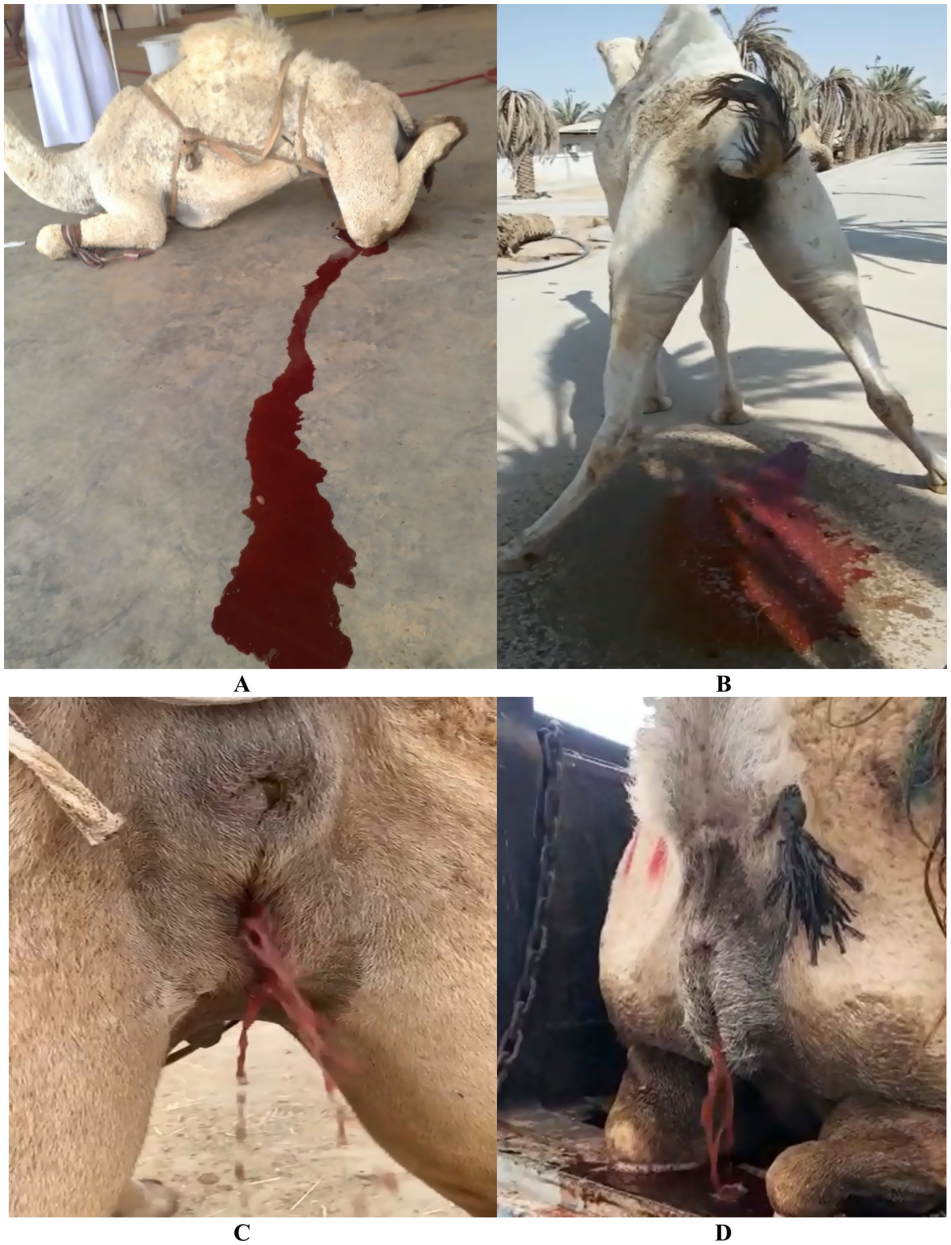
Clinical signs of red urine in camels suggestive of urinary tract disorders. **(A–D)** Varying degrees of red urine in males and females, with associated discomfort post-urination. These signs may indicate upper urinary tract infections such as pyelonephritis. Adapted from (5).

**Figure 7:**
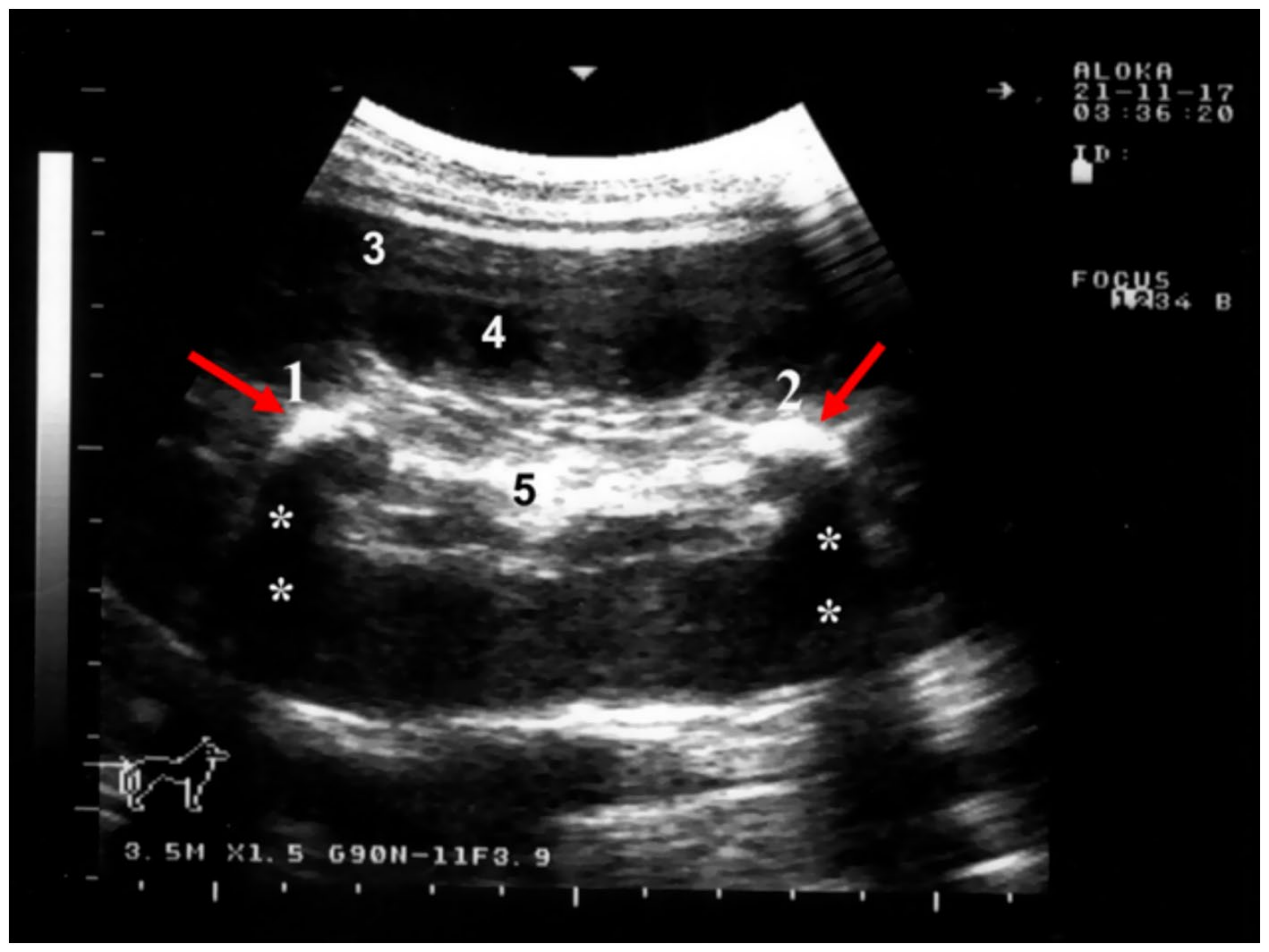
Ultrasonographic image of nephrolithiasis in a female camel with chronic hematuria. Echogenic renal stones with acoustic shadowing are visible, confirming nephrolith presence and highlighting ultrasonography’s role in diagnosing persistent urinary symptoms. Adapted from (3).

Ultrasonography has proven to be an effective, non-invasive diagnostic tool for detecting urinary calculi in dromedary camels (8). The incidence of urolithiasis is notably high, particularly in male camels fed concentrated diets, often resulting in obstructive uropathy and urinary tract infections (4). Sonography enables visualization of calculi throughout the urinary tract—from the kidneys to the penile body—characterized by strong echogenicity with distal acoustic shadowing. Studies have demonstrated that ultrasound allows veterinarians to detect both small and large stones in real time, providing critical information on their size, location, and impact on the urinary system (4). Due to its high sensitivity and capacity to guide clinical management, ultrasonography is considered a fundamental tool for diagnosis and monitoring of urinary calculi in camels. Calculi in the kidneys appear as echogenic foci with distal shadowing, while transrectal imaging of the urinary bladder reveals homogenous or focal echogenic deposits (Figures 6, 7) (5). Nephrolithiasis may be visualized as similar echogenic deposits within one or both kidneys.

### 4.3 Urine retention, dribbling, and anuria in dromedary camels

In dromedaries, urine retention, dribbling, and anuria can result from obstructive or neurological etiologies. Urine retention in dromedary camels may be partial or complete, manifesting as urine dribbling or anuria. Causes include urinary tract calculi, bladder paralysis, penile fracture, hematoma, balanitis, urinary neoplasia, and severe dehydration (3). Urine dribbling is characterized by involuntary leakage of small amounts of urine, often due to urinary tract infections, bladder dysfunction, physical obstructions such as calculi, or impaired bladder control resulting from nerve damage or infection (4).

**Figure 8:**
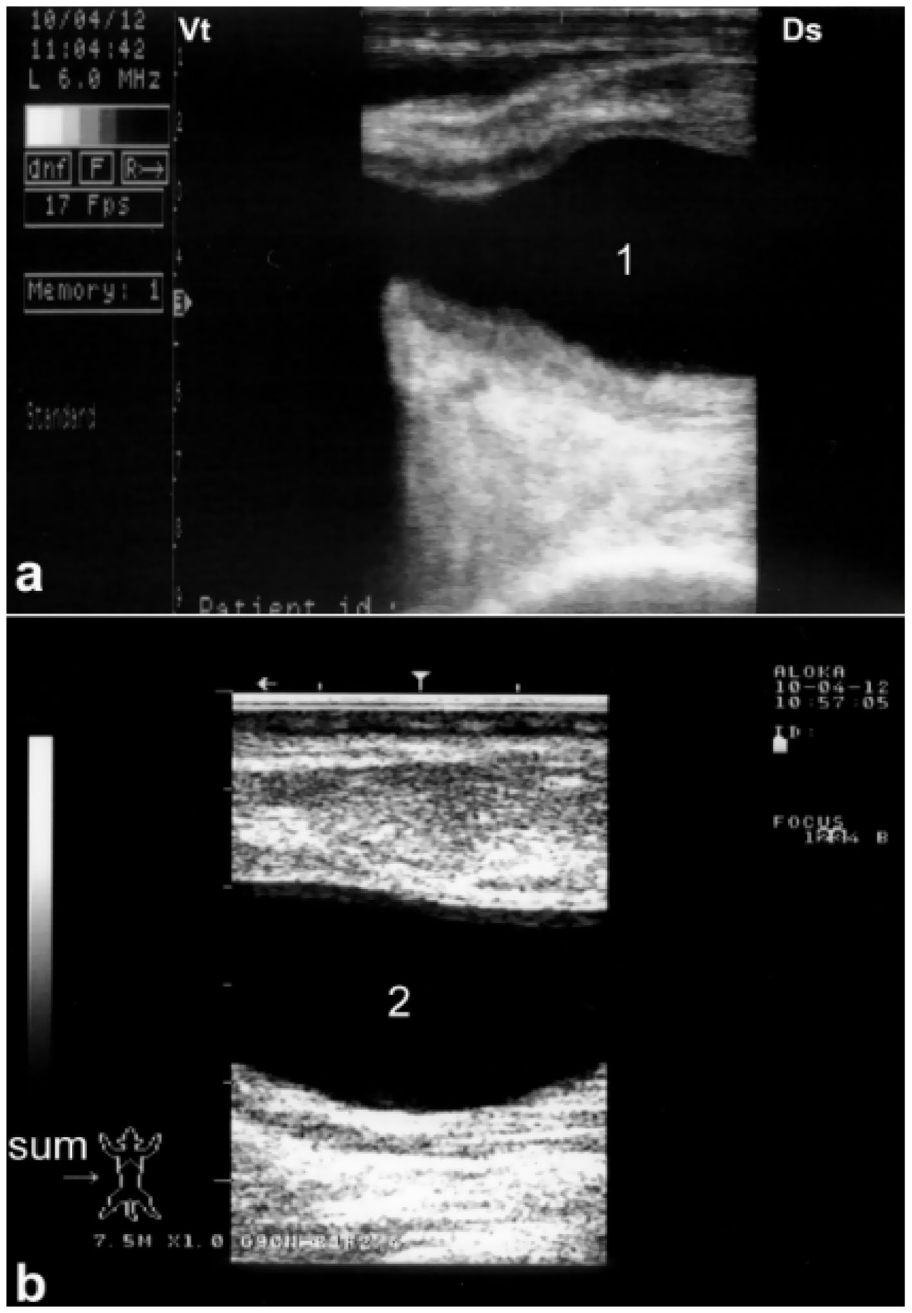
Sonographic detection of urethral dilation in a male camel with chronic urine dribbling. **(a)** Dilated pelvic urethra; **(b)** dilated penile urethra. Findings suggest partial obstruction, with implications for ascending infections and renal complications. Adapted from (4).

Physiological camels’ adaptation to desert environments allows them to survive prolonged water deprivation; however, in severe dehydration, renal urine production may cease as a water conservation mechanism (4). Anuria may also arise from renal injury or infection impairing kidney function. While potentially life-threatening, anuria is often reversible with prompt rehydration and treatment. Untreated cases can lead to severe complications (5). Sonographically, camels with urine retention exhibit a markedly distended urinary bladder, altered bladder wall thickness, back pressure effects and early hydronephrosis, significant dilation of the pelvic, and penile urethra (Figure 8). Mild dilation of the renal sinus may also be observed. Tube cystotomy is employed for treatment in cases of anuria with ruptured urethra or a distended but intact bladder. Surgical management involves draining subcutaneous urine via multiple latero-ventral abdominal incisions, performing a post-scrotal urethrostomy with an indwelling catheter, and conducting a cystotomy with catheterization. Surgical management includes drainage of subcutaneous urine through multiple skin incisions on the latero-ventral abdominal wall, post-scrotal urethrostomy with an indwelling catheter, and cystotomy with catheterization (37).

**Figure 9:**
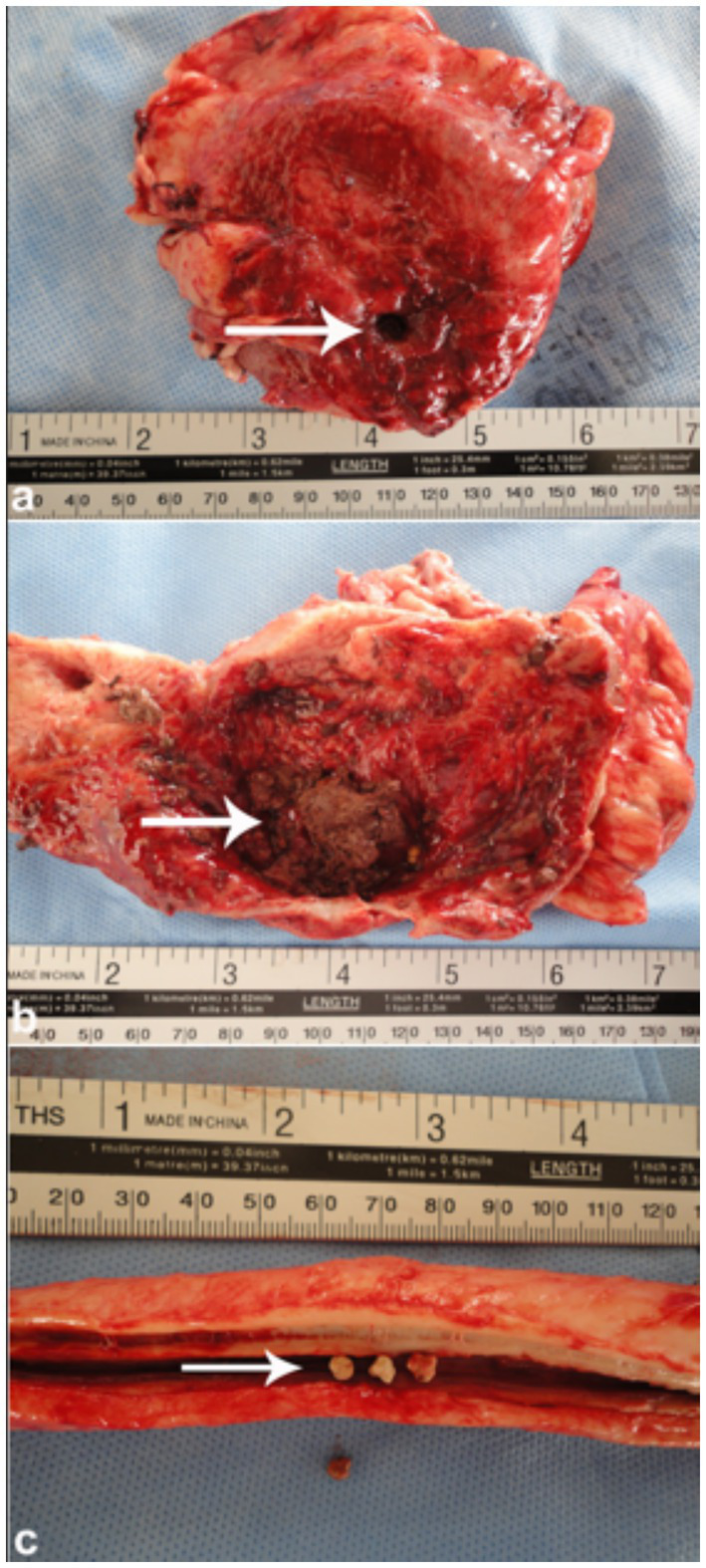
Necropsy of a camel with bladder rupture. **(a)** Perforated bladder wall; **(b)** presence of blood clots and a urolith; **(c)** multiple stones in the penile body. These findings show severe outcomes of obstructive urolithiasis. Adapted from (4).

**Figure 10:**
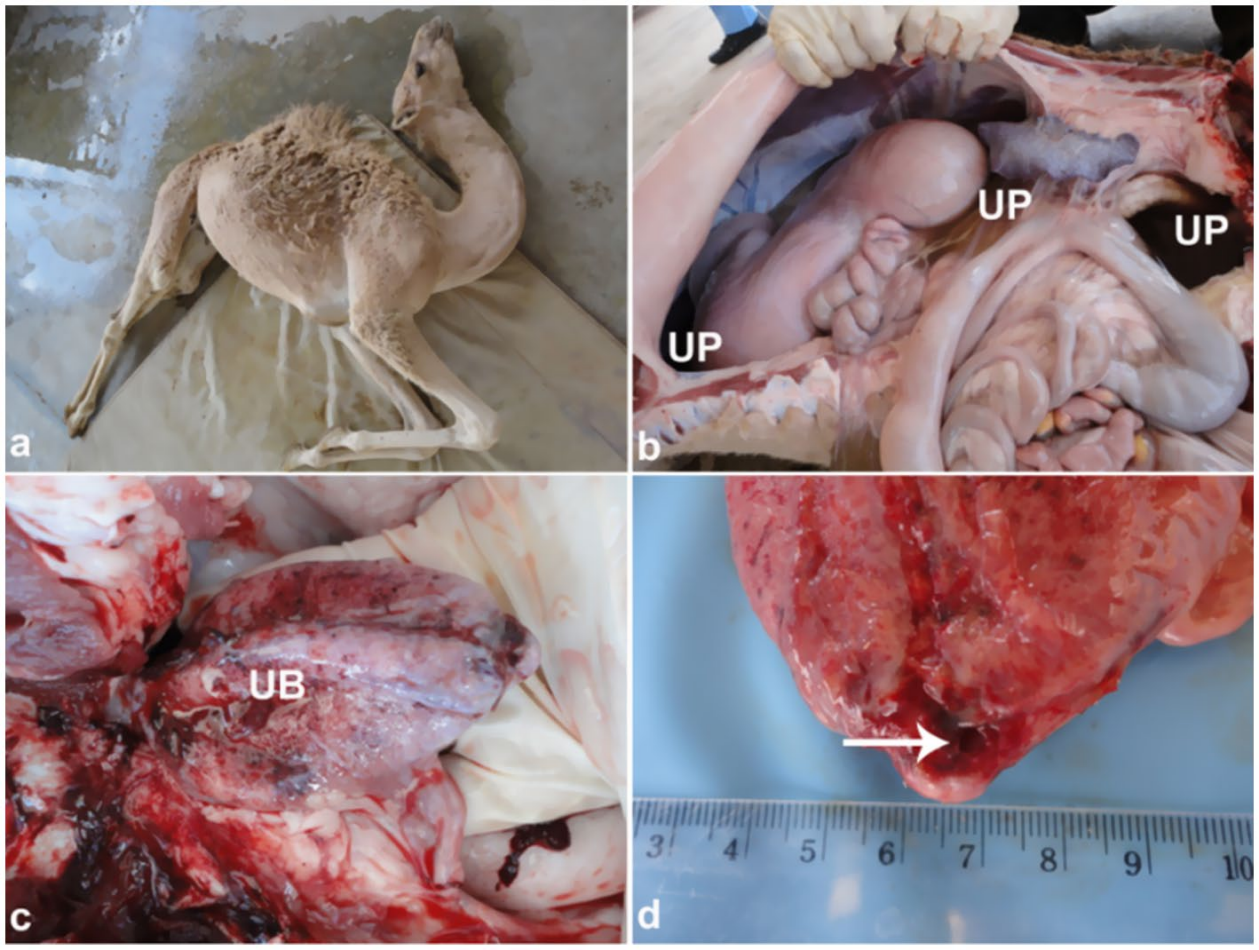
Clinical and necropsy findings in a camel calf with bladder rupture. **(a)** Abdominal distension; **(b)** uroperitoneum; **(c)** hemorrhagic bladder serosa; **(d)** bladder wall perforation. Emphasizes importance of early detection of urinary tract rupture. Adapted from (4).

Postmortem examination typically reveals a collapsed urinary bladder with a ruptured wall. The bladder may contain blood clots or hematuria-associated debris consistent with blood clots, as well as urinary stones, which can also be found within the penile body (Figure 9). Necropsy findings often include uroperitoneum that can be confirmed antemortem during ultrasound-guided abdominocentesis. Other postmortem findings may include congested and hemorrhagic bladder serosa, and perforation of the bladder wall (Figure 10).

### 4.4 Rupture of the urinary bladder

**Figure 11:**
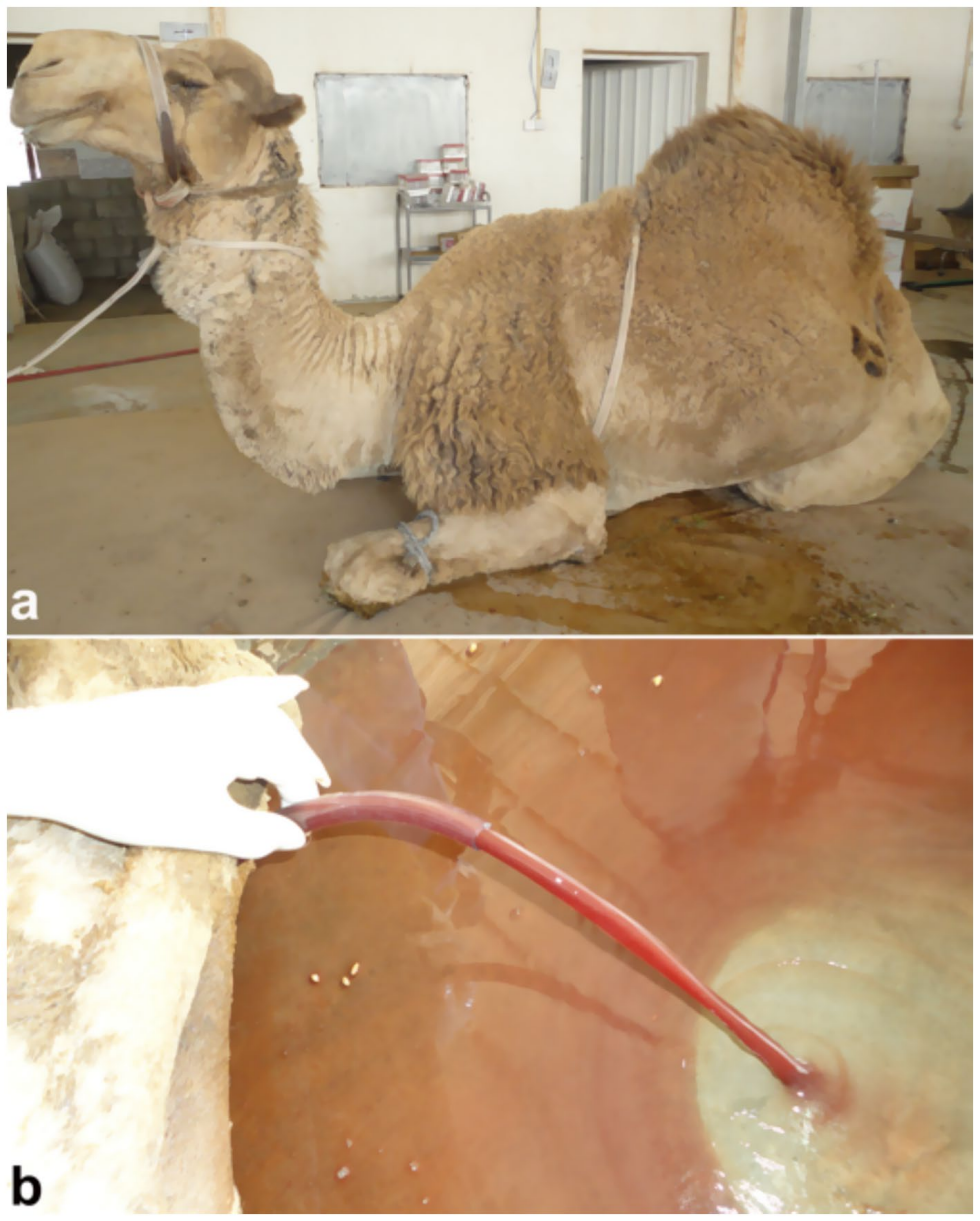
Laparotomy image of a male camel with urinary retention due to obstructive urolithiasis. Large volume of reddish urine indicates hemorrhage and bladder pressure buildup, underlining need for timely surgical intervention. Adapted from (4).

Ultrasound is a highly valuable, non-invasive diagnostic tool for confirming urinary bladder rupture in dromedaries, enabling prompt detection and assessment of this potentially life-threatening condition (1). Bladder rupture secondary to urethral obstruction has been reported in camelids, with two cases documented in the literature (33, 38, 39). Sonographically, camels with ruptured bladders typically present with uroperitoneum, visible as anechoic free fluid within the peritoneal cavity. Differential diagnosis of free peritoneal fluid includes peritoneal effusions due to other causes such as peritonitis. In addition, loss of normal bladder contour is a fundamental sonographic finding. Abdominal organs such as stomach compartments, intestines, liver, kidneys, and spleen can be observed floating in the effusion (4). The bladder often appears collapsed with a thickened, corrugated mucosa. Clinically, large volumes of hemorrhagic peritoneal fluid may be collected (Figure 11).

**Figure 12:**
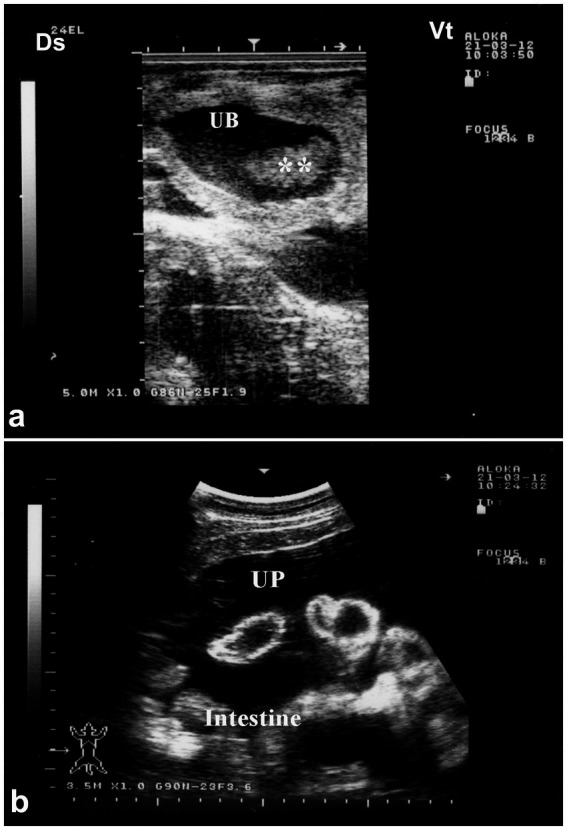
Ultrasonographic features of bladder rupture. **(a)** Collapsed bladder with blood clots; **(b)** free abdominal fluid (uroperitoneum) with floating intestines. Highlights key sonographic signs of urinary leakage. Adapted from (1).

Ultrasound enables clinicians to assess the extent of urinary bladder rupture, distinguishing between partial and complete tears, and to identify associated complications such as peritonitis (4). Consequently, sonography is an invaluable tool for confirming bladder rupture, guiding treatment decisions, and monitoring recovery in affected dromedaries. Sonographically, extensive uroperitoneum is evident, with abdominal organs—including the liver and intestines—seen floating within the fluid (Figure 12). In severe cases, the kidneys, spleen, and fibrinous material may also be visualized suspended in the uroperitoneum. Transrectal ultrasonography further reveals the collapsed urinary bladder with markedly thickened walls, floating within the uroperitoneal fluid.

### 4.5 Rupture of the urethra

**Figure 13:**
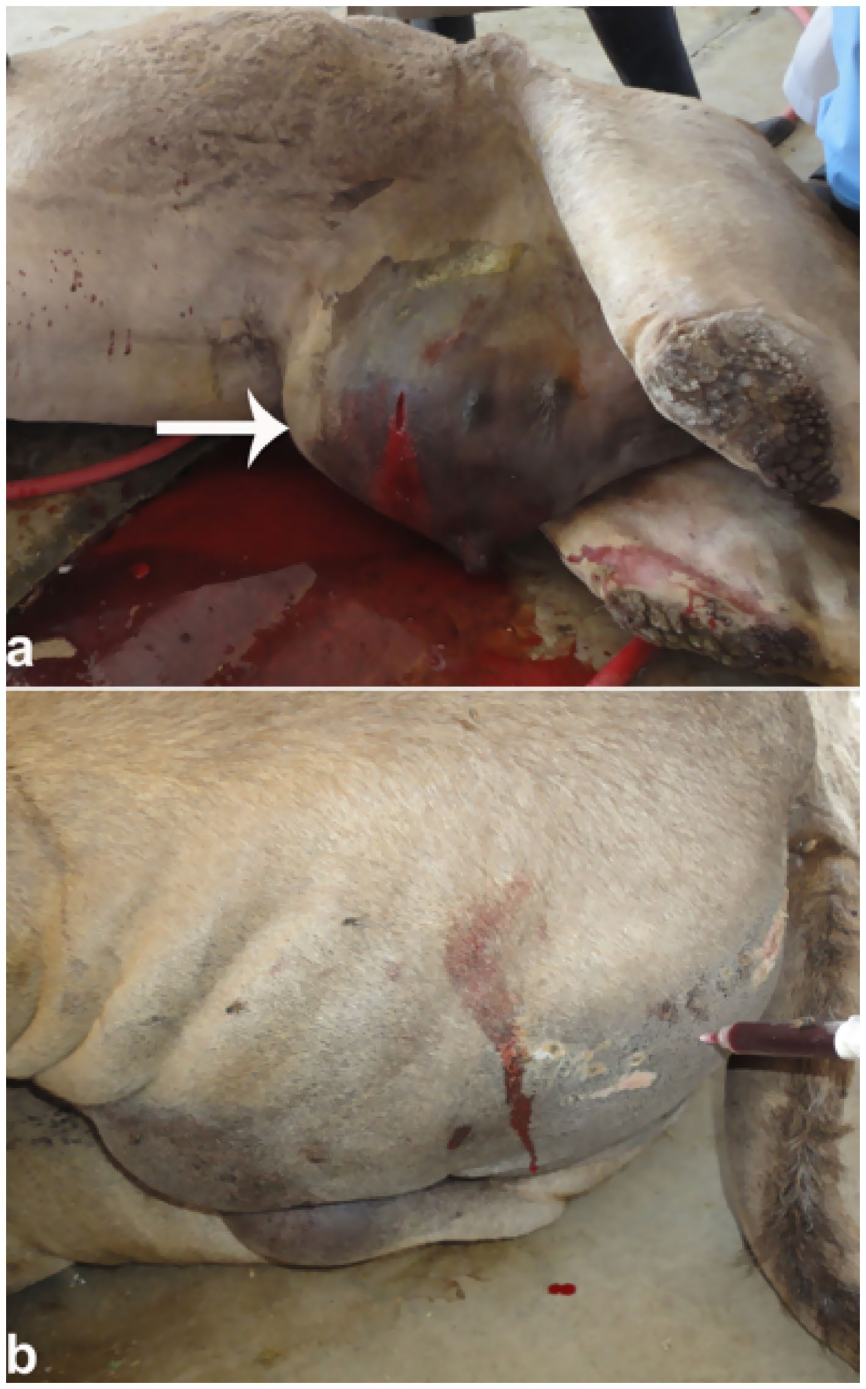
Urethral rupture in a male camel. **(a)** Penile edema with red urine release upon puncture; **(b)** urine infiltration in gluteal muscles. Supports diagnosis of obstructive uropathy and emphasizes need for early intervention. Adapted from (4).

In male dromedaries, urethral perforation commonly results from trauma or obstructive uropathy (Figure 13). Sonographic examination can identify urethral abnormalities and urine leakage into surrounding tissues, confirming rupture (4). Sonography may also reveal subcutaneous fluid tracking or gas if infection is present. In addition, ultrasonography precisely localizes the rupture site, aiding clinical decisions between medical management and surgical repair. This imaging modality is essential for accurate and timely diagnosis of urethral perforation in dromedaries, contributing to improved clinical outcomes (1, 4). Sonographically, although rare, the urinary bladder wall remains intact but may contain hyperechoic, non-mobile deposits. The penile body often appears grossly enlarged, with urine detectable in adjacent tissues, and the obstructing lesion can be visualized causing partial or complete urethral blockage.

### 4.6 Inflammation of the urinary bladder

Cystitis, inflammation of the bladder primarily caused by bacterial infections or urinary tract obstruction, can be effectively assessed by sonography, which reveals changes in the bladder wall and its contents (3). Sonographically, cystitis often appears with diffuse or focal wall thickening and may produce sediment. The bladder wall appears thickened with irregularities on the inner surface, similar to findings reported in canines and felines (40–42). To preserve renal function in dromedary camels, early detection of cystitis by ultrasound and prompt antimicrobial treatment are essential to prevent complications like ascending pyelonephritis and allow timely intervention (1).

**Figure 14:**
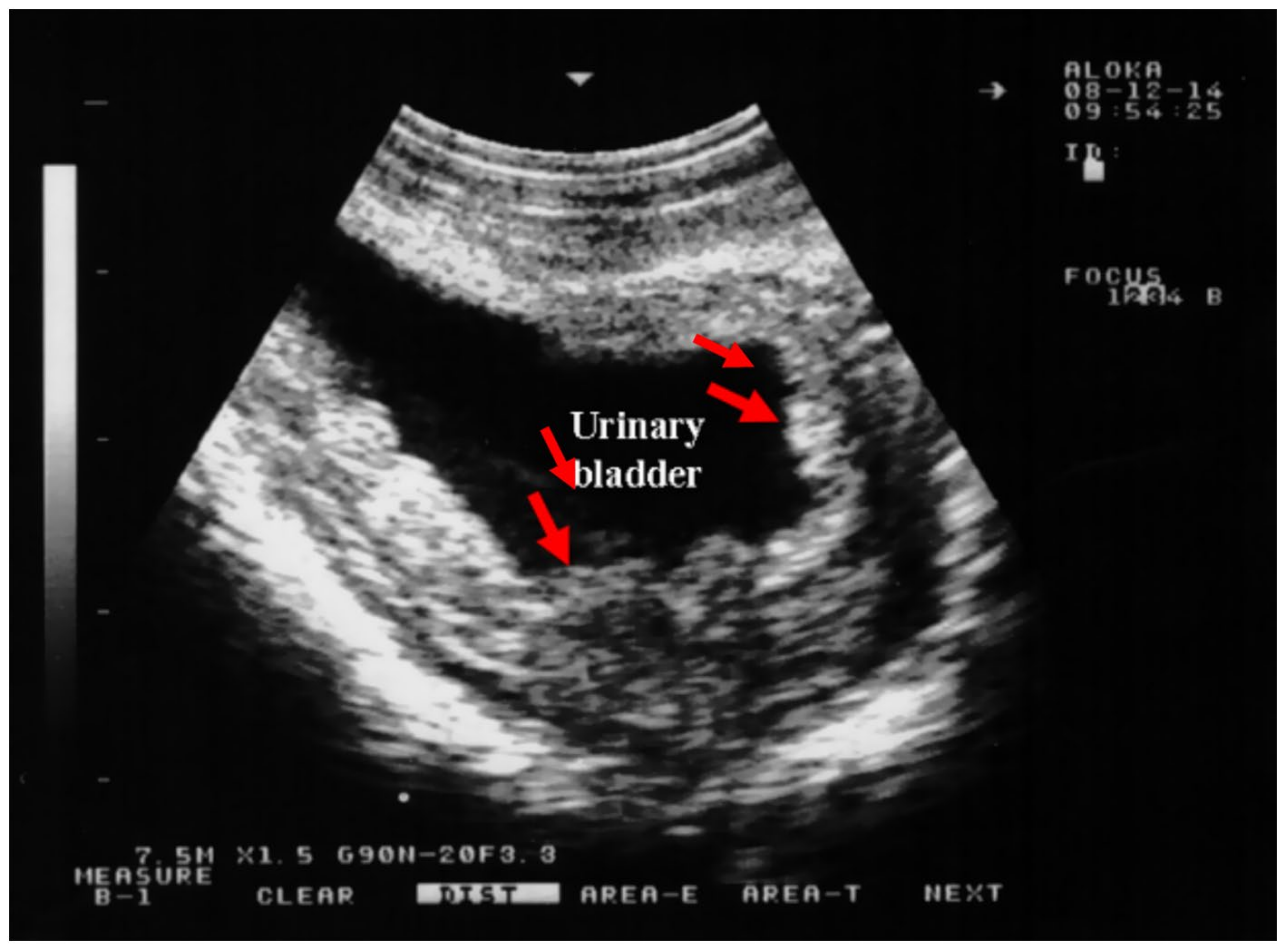
Transrectal ultrasound showing cystitis in a female camel. Narrowed bladder lumen with thickened, corrugated mucosa indicates infection. Useful in early detection of lower urinary tract infections. Adapted from (1).

An abattoir study of camel urine showed that 54.5% of samples contained *Staphylococcus* spp., 27.3% *Corynebacterium* spp., and 18.2% *Escherichia coli* (10). In camels with bladder inflammation, ultrasound reveals a thickened, corrugated bladder wall, often accompanied by enlarged regional lymph nodes. Echogenic deposits, typically hyperechoic, are frequently observed in the ventral bladder region and, in severe cases, may be diffusely distributed throughout the bladder lumen (Figure 14).

### 4.7 Urinary neoplasia

Neoplasia of the urinary system, either benign or malignant, although relatively rare in dromedary camels, can present as irregular masses with heterogeneous echogenicity and altered renal or bladder architecture (2). On ultrasound, renal neoplasms such as renal cell carcinoma typically appear as well-defined or irregularly shaped masses with variable echogenicity (2). Ultrasonographic imaging also aids in assessing the extent of local invasion or metastasis, providing valuable information to guide treatment strategies when available. Color Doppler may help assess vascularization for malignancy suspicion. Given its ability to detect soft tissue abnormalities and characterize tumors in real time, renal ultrasonography is a vital tool for early detection and management of urinary tract neoplasia in dromedary camels, improving clinical outcomes.

**Figure 15:**
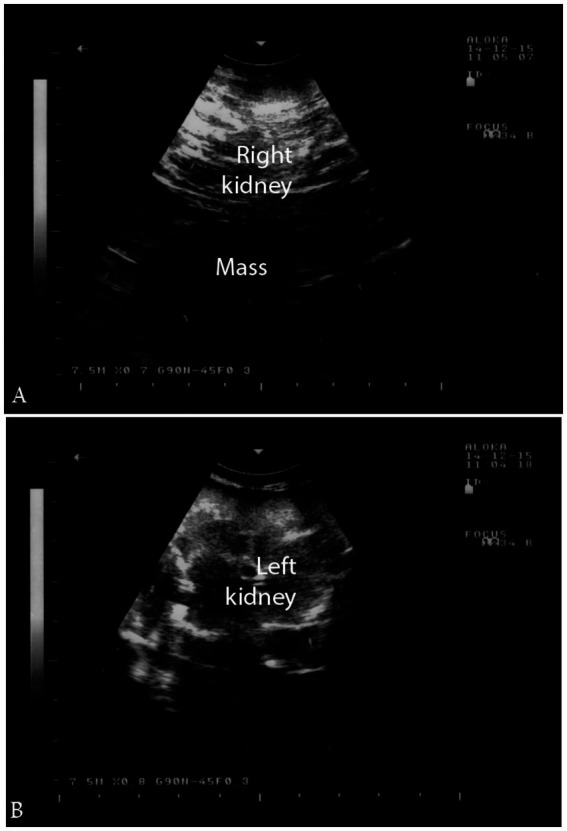
Ultrasonographic findings of renal cell carcinoma. **(a)** Hypoechoic mass in the right kidney; **(b)** normal left kidney. Helps distinguish tumors from inflammatory conditions and supports early diagnosis. Adapted from (2).

In a confirmed case of right kidney renal cell carcinoma, transrectal ultrasonography revealed a large, irregularly shaped, hypoechoic, cavitated mass extending caudally within the right renal parenchyma, while the left kidney appeared normal on subjective assessment (Figure 15). These ultrasound findings await confirmation via post-mortem examination. Histological analysis of the renal tissue confirmed renal cell carcinoma with tubular differentiation, malignant epithelial lining, and nuclear anaplasia (2).

## 5 Limitations, diagnostic challenges, and future prospects

Ultrasonography is a valuable, non-invasive tool for diagnosing urinary tract disorders in dromedary camels, and is currently the most accessible modality in field settings. This procedure has been frequently used to evaluate dromedaries with thoracic conditions (43), gastrointestinal issues (44–49), abdominal masses and neoplasia (50–54), as well as hepatic disorders (55, 56). Ultrasound has also proven effective in detecting and aiding the control of certain highly contagious infectious diseases in dromedaries (57–59). Additionally, this imaging modality has been utilized at camel beauty festivals to identify cosmetic procedures such as filler injections and various forms of tampering (60–62). However, several limitations impact its clinical utility still exist. Variability in operator experience, lack of standardized protocols specific to camelid anatomy, and disparities in equipment quality contribute to inconsistent image interpretation. These factors, along with the potential for false positives—such as mistaking gas shadows or intestinal loops for calculi—and false negatives—such as missing early-stage inflammation or small uroliths due to poor image resolution—underscore the importance of correlating imaging findings with clinical signs and, where feasible, histopathological data. Accessibility to portable ultrasound units remains a challenge, especially in remote regions. Future research should focus on standardizing sonographic criteria and validating imaging markers through longitudinal studies correlating ultrasonographic findings with clinical outcomes and treatment responses. Advancements in imaging modalities—such as Doppler, contrast-enhanced, and 3D ultrasonography—offer promising avenues for improved visualization of urinary tract pathology. However, doppler and contrast enhanced ultrasonography are not commonly available in the field. In addition, telemedicine may enable remote diagnostics in dromedaries, allowing timely assessment and management of health issues in remote areas. Furthermore, studies exploring epidemiological trends, risk factors, and genetic predispositions may enhance disease prevention and management strategies. Building collaborative databases and fostering interdisciplinary research will be essential to refine diagnostic accuracy and broaden the clinical applicability of ultrasound in camelid medicine. Finally, investigating possible nutritional or mineral imbalances that may lead to calculi formation or cystitis in dromedaries is also recommended.

## 6 Conclusion

Urinary tract disorders are being reported with increasing frequency in dromedary camels. Ultrasonography has become a central, non-invasive diagnostic modality that significantly enhances clinical assessment and understanding of these disorders. As a real-time imaging technique, it provides detailed visualization of a wide spectrum of urinary tract abnormalities—including pyelonephritis, renal abscesses, urinary calculi, urine retention, bladder and urethral ruptures, cystitis, and urinary neoplasms. This review underscores the critical role of ultrasonography in accurately identifying and differentiating these conditions based on their distinct sonographic features. With correct interpretation, ultrasonographic findings can guide timely clinical decisions and therapeutic interventions. Ongoing research and clinical reporting will be essential for refining imaging criteria and enhancing diagnostic precision. By presenting recent research findings, this review seeks to raise awareness and guide future approaches for effectively managing urinary tract diseases, ultimately contributing to improved health outcomes in both domestic and wild camel populations. Encouraging wider adoption of ultrasonography, along with targeted training for field veterinarians and camel caregivers, is strongly recommended to facilitate early diagnosis, improve treatment outcomes, and support ongoing surveillance efforts.

## References

[1] Tharwat M Al-Sobayil F. Ultrasonographic findings in camels (*Camelus dromedarius*) with different urinary affections. J Camel Pract Res. (2016) 23:301–8. doi: 10.5958/2277-8934.2016.00050.3

[2] Tharwat M Al-Sobayil F Ali A Derar D Khodeir M. Renal cell carcinoma in a female Arabian camel: clinical, hematobiochemical, ultrasonographic and pathologic findings. J Camel Pract Res. (2017) 24:61–6. doi: 10.5958/2277-8934.2017.00009.1

[3] Tharwat M. Ultrasonography of the kidneys in healthy and diseased camels (*Camelus dromedarius*). Vet Med Intern. (2020) 2020:7814927. doi: 10.1155/2020/7814927 PMC7596541 33144934

[4] Tharwat M. Obstructive urolithiasis in dromedary camels: clinical, ultrasonographic and postmortem findings. J Camel Pract Res. (2021) 28:85–93. doi: 10.5958/2277-8934.2021.00013.8

[5] Tharwat M Elmoghazy HMM Almundarij TI. Red urine syndrome in dromedary camels: clinical, etiological, hematobiochemical, sonographic, and pathologic findings. Open Vet J. (2024) 14:2463–74. doi: 10.5455/OVJ.2024.v14.i9.35, PMID: 39553784 PMC11563604

[6] Tharwat M Elmoghazy HMM Saeed EMA Alkheraif AA. Renal abscessation in dromedary camels: clinical, ultrasonographic, hematobiochemical, and etiological investigations. Open Vet J. (2025) 15:1289–303. doi: 10.5455/OVJ.2025.v15.i3.20, PMID: 40276189 PMC12017707

[7] Kojouri GA Nourani H Sadeghian S Imani H Raisi A. Pathological findings of slaughtered camels’ (Camelus dromedaries) kidneys in Najaf-abad, Iran. Vet Res Forum. (2014) 5:231–5. 25568724 PMC4279641

[8] Saini K Dadhich H Mathur M Tripathi A. Histopathological studies on renal lesions in dromedary camel (Camelus dromedaries). J Camel Pract Res. (2015) 22:113–9. doi: 10.5958/2277-8934.2015.00018.1

[9] Barakat SEM Hizab FA Moqbel MS. Pathological and serobiochemical studies on naturally occurring kidney affections in camels (Camelus dromedaries). J Camel Pract Res. (2017) 24:55–9. doi: 10.5958/2277-8934.2017.00008.x

[10] Khamis GF Abd Ellah MR Elnisr NA Abd Elmoety M Manaa AM Aamer AA. Bacteriological and qualitative analysis of camel's urine and its relation to urinary bladder pathological changes. Assiut Vet Med J. (2009) 55:1–12.

[11] Abdeldjalil D. Acquired obstructive urolithiasis in male *Camelus dromedarius* from Southeast Algeria: report of 11 cases. J Camelid Sci. (2020) 13:56–65.

[12] Ali A. Observations on the topography of the reproductive tract of the Arabian female camel. J Agricul Vet Sci, Qassim University. (2010) 3:33–42.

[13] Jones ML Miesner MD. Urolithiasis In: Anderson DE Rings DM, editors. Food animal practice. Fifth ed. Saint Louis: W.B. Saun ders (2009). 322–5.

[14] Smith B. P. Large animal internal medicine. 4th Edn. Mosby Elsevier, USA. (2009). pp. 415–1024.

[15] Sharun K Manjusha KM Kumar R Pawde AM Malik YP Kinjavdekar P. Prevalence of obstructive urolithiasis in domestic animals: an interplay between seasonal predisposition and dietary imbalance. Iraqi J Vet Sci. (2021) 35:227–32. doi: 10.33899/ijvs.2020.126662.1358

[16] Sickinger M Windhorst A. A systematic review on urolithiasis in small ruminants according to nutrition-dependent prevalence and outcome after surgery. Vet World. (2022) 15:809–17. doi: 10.14202/vetworld.2022.809-817, PMID: 35497951 PMC9047136

[17] Gutierrez C Corbera JA Doreste F Padrón TR Morales M. Silica urolithia sis in the dromedary camel in a subtropical climate. Vet Res Commun. (2002) 26:437–42. doi: 10.1023/A:1020534323968, PMID: 12241096

[18] Gutierrez C Padrón M Bañares A Palacios MP. Urinary retention in two male dromedaries due to silica uroliths. Zentralbl Veterinarmed A. (1999) 46:523–6. doi: 10.1046/j.1439-0442.1999.00235.x, PMID: 10605361

[19] Ewoldt JM Jones ML Miesner MD. Surgery of obstructive urolithiasis in ruminants. Vet Clin North Am Food Anim Pract. (2008) 24:455–65. doi: 10.1016/j.cvfa.2008.06.003, PMID: 18929952

[20] Makhdoomi DM Gazi M. Obstructive urolithiasis in ruminants – a review. Vet World. (2013) 6:233. doi: 10.5455/vetworld.2013.233-238

[21] Smith JA Divers TJ Lamp TM. Ruptured urinary bladder in a post-parturient cow. Cornell Vet. (1983) 73:3–12. 6825450

[22] Kock RA. Obstructive urethral calculi in the male camel: report of two cases. Vet Rec. (1985) 117:494–6. 4082397 10.1136/vr.117.19.494

[23] Rosser JM Jacob SI Brounts SH. Use of tube cystostomy in the surgical management of obstructive urolithiasis in a bactrian camel. J Am Vet Med Assoc. (2019) 254:868–73. doi: 10.2460/javma.254.7.868, PMID: 30888274

[24] Videla R van Amstel S. Urolithiasis. Vet Clin North Am Food Anim Pract. (2016) 32:687–700. doi: 10.1016/j.cvfa.2016.05.010, PMID: 27719765

[25] Ermilio EM Smith MC. Treatment of emergency conditions in sheep and goats. Vet Clin North Am Food Anim Pract. (2011) 27:33–45. doi: 10.1016/j.cvfa.2010.10.005, PMID: 21215888

[26] Singh KB Rao SV. Pelvic urethrotomies in bulls. Vet Rec. (1979) 105:137–41. doi: 10.1136/vr.105.7.137, PMID: 552747

[27] Oman RE Reppert EJ Streeter RN Jones M. Outcome and complications in goats treated by perineal urethrostomy for obstructive urolithiasis: 25 cases (2010–2017). J Vet Intern Med. (2019) 33:292–6. doi: 10.1111/jvim.15360, PMID: 30499606 PMC6335529

[28] Bhadwal M Sudhan NA. Retention of urine in camel (2000) 77:242–3.

[29] Jones ML Gibbons PM Roussel AJ Dominguez BJ. Mineral composition of uroliths obtained from sheep and goats with obstructive urolithiasis. J Vet Intern Med. (2017) 31:1202–8. doi: 10.1111/jvim.14743, PMID: 28556535 PMC5508333

[30] Cowley J Hopper R. Management of urolithiasis in breeding bulls. Clin Theriogenol. (2023) 15:5–10. doi: 10.58292/ct.v15.9646

[31] Mobarak AM el-Wishy AB Samira MF. The penis and prepuce of the one-humped camel (*Camelus dromedarius*). Zentralbl Veterinarmed A. (1972) 19:787–95. doi: 10.1111/j.1439-0442.1972.tb00532.x, PMID: 4629466

[32] Degen AA Lee DG. The male genital tract of the dromedry (one-humped) camel (*Camelus dromedarius*): gross and microscopic anatomy. Anat Histol Embryol. (1982) 11:267–82. doi: 10.1111/j.1439-0264.1982.tb00995.x, PMID: 6216826

[33] Carlos GJA Corbera FB. Obstructive phosphate urolithiasis in a dromedary camel: a case report. J Camel Pract Res. (2008) 15:1–5.

[34] Van Metre DC Divers TJ. Diseases of the renal system: urolithiasis In: Smith BP, editor. Large animal internal medicine. 3rd ed. St. Louis, MO: Mosby (2002). 853–60.

[35] Ewoldt JI Baird AN Anderson DE. Urogenital surgery in camelids In: Cebra C Anderson DE Tibary A, editors. Lama and alpaca care. St. Louis: Elsevier (2014). 702–9.

[36] Khaki Z. Khazraeinia P. Bokaei S. (2006) “Prevalence rate of bladder stones in slaughtered camel around Tehran.” In: *Proceedings of the 14th Iranian veterinary congress in Tehran, Iran*, 250–251.

[37] Kaswan BL Tanwar M Purohit NR Gahlot TK. Surgical management of urinary retention in three camels (*Camelus dromedarius*). J Camel Pract Res. (2015) 22:141–4. doi: 10.5958/2277-8934.2015.00022.3

[38] McLaughlin BG Evans NC. Urethral obstruction in a male llama. J Am Vet Med Assoc. (1989) 195:1601–2. 2599947

[39] Dart AJ Dart CM Hodgson DR. Surgical management of a ruptured bladder secondary to a urethral obstruction in an alpaca. Aust Vet J. (1997) 75:793–5. 9404609 10.1111/j.1751-0813.1997.tb15653.x

[40] Conze T. Rezidivierende vaginitis und Zystitis aufgrund einer vagina und Zervix duplex bei einer Hündin – ein Fallbericht [recurrent vaginitis and cystitis due to a vagina and cervix duplex in a bitch - a case report]. Tierarztl Prax Ausg K Kleintiere Heimtiere. (2023) 51:285–90. doi: 10.1055/a-2122-5408 37820621

[41] Labelle O Penninck D Butty EM Hahn S Dunn M. Pseudomembranous cystitis in cats with presumed or confirmed mineralization: a retrospective study of 26 cases (2016-2021). J Vet Intern Med. (2023) 37:1806–14. doi: 10.1111/jvim.16819, PMID: 37497780 PMC10472995

[42] Moreau M Haudiquet P Monton C Arnault F Jossier R. Ultrasound diagnosis of cystic cystitis with von Brunn's nest in two cats. JFMS Open Rep. (2024) 10:20551169241298745. doi: 10.1177/20551169241298745, PMID: 39734662 PMC11672381

[43] Tharwat M Al-Sobayil F. Ultrasonographic findings in camel calves (*Camelus dromedarius*) with thoracic affections. J Camel Pract Res. (2016) 23:287–90. doi: 10.5958/2277-8934.2016.00048.5

[44] Tharwat M. Chronic peritonitis in dromedary camels: clinical, hematobiochemical, ultrasonographic and pathologic findings. J Camel Pract Res. (2019) 26:169–72. doi: 10.5958/2277-8934.2019.00026.2

[45] Tharwat M. Ultrasonography of the abdomen in healthy and diseased camels (Camelus dromedaries). J Appl Anim Res. (2020) 48:300–12. doi: 10.1080/09712119.2020.1788035 PMC7192722 32101826

[46] Tharwat M Alkhedhairi S Marzok M. Intestinal obstruction in dromedary camels: clinical and ultrasonographic findings as well as variations in acid-base balance, blood gases and hematobiochemical profiles. IJAB. (2024) 13:500–4. doi: 10.47278/journal.ijab/2024.131

[47] Tharwat M Al-Sobayil F. Ultrasonographic findings in camels (*Camelus dromedarius*) with abdominal disorders. J Camel Pract Res. (2016) 23:291–9. doi: 10.5958/2277-8934.2016.00049.7

[48] Tharwat M Al-Sobayil F Ali A Buczinski S. Ultrasonographic evaluation of abdominal distension in 52 camels (*Camelus dromedarius*). Res Vet Sci. (2012) 93:448–56. doi: 10.1016/j.rvsc.2011.07.009, PMID: 21840025

[49] Tharwat M El-Ghareeb WR Almundarij TI. Depraved appetite in dromedary camels: clinical, ultrasonographic, and postmortem findings. Open Vet J. (2024) 14:652–63. doi: 10.5455/OVJ.2024.v14.i2.5, PMID: 38549572 PMC10970125

[50] Sadan M Tharwat M Alkhedhairi S Refaai W Moghazy HMEL Khodier MM. Abdominal pedunculated leiomyoma in a male dromedary camel: clinical, hematobiochemical, ultrasonographic and pathologic findings. Int J Vet Sci. (2024) 13:458–62. doi: 10.47278/journal.ijvs/2023.114

[51] Tharwat M El-Shafaey E Alkheraif AA. Ultrasonographic evaluation of thoracic and abdominal neoplasia in domestic ruminants: a systemic review. Open Vet J. (2024) 14:1751–60. doi: 10.5455/OVJ.2024.v14.i8.2 39308737 PMC11415916

[52] Tharwat M Alkheraif AA Elmoghazy HMM Haridy M Marzok M. Disseminated pyogranulomas in a female dromedary camel: hematobiochemical, sonographic and pathologic investigations. Int J Vet Sci. (2025) 14:120–4. doi: 10.47278/journal.ijvs/2024.221

[53] Tharwat M El-Shafaey E Sadan M Ali A Al-Sobayil F Al-Hawas A. Omaso-abomasal adenocarcinoma in a female Arabian camel (*Camelus dromedarius*). J Appl Anim Res. (2018) 46:1268–71. doi: 10.1080/09712119.2018.1495644

[54] Tharwat M Haridy M Elmoghazy HMM Elnahas A Alkheraif AA. Abdominal fat necrosis in a female dromedary camel: clinical, hematobiochemical, sonographic, and pathologic findings. Open Vet J. (2024) 14:1726–32. doi: 10.5455/OVJ.2024.v14.i7.22, PMID: 39175969 PMC11338617

[55] Tharwat M El Moghazy HM Oikawa S. Ultrasonographic verification of hepatic hydatidosis in a female dromedary camel: a case report. J Vet Med Sci. (2023) 85:1286–90. doi: 10.1292/jvms.23-0325, PMID: 37880080 PMC10788162

[56] Tharwat M. Ultrasonography of the liver in healthy and diseased camels (Camelus dromedaries). J Vet Med Sci. (2020) 82:399–407. doi: 10.1292/jvms.19-0690, PMID: 32101826 PMC7192722

[57] Tharwat M Tsuka T. Diagnostic utility of ultrasonography for thoracic and abdominal bacterial and parasitic diseases in ruminants: a comprehensive overview. Front Vet Sci. (2024) 11:1435395. doi: 10.3389/fvets.2024.1435395, PMID: 39286596 PMC11402809

[58] Tharwat M Ali H Alkheraif AA. Clinical insights on paratuberculosis in Arabian camels (*Camelus dromedarius*): a review. Open Vet J. (2025) 15:8–17. doi: 10.5455/OVJ.2025.v15.i1.2, PMID: 40092173 PMC11910307

[59] Tharwat M Al-Sobayil F Ali A Hashad M Buczinski S. Clinical, ultrasonographic, and pathologic findings in 70 camels (*Camelus dromedarius*) with Johne’s disease. Can Vet J. (2012) 53:543–8. 23115369 PMC3327595

[60] Tharwat M Al-Hawas A. Ultrasound detection of cosmic fillers injection of lips in camel beauty pageants: first report in veterinary medicine. Trop Anim Health Prod. (2021) 53:53. doi: 10.1007/s11250-020-02551-9, PMID: 33387053

[61] Tharwat M Sadan M Al-Hawas A. The emerging topic of injected cosmetic fillers in the perinasal region of dromedary camels: ultrasonographic and radiographic verification. Open Vet J. (2024) 14:840–5. doi: 10.5455/OVJ.2024.v14.i3.11, PMID: 38682143 PMC11052611

[62] Tharwat M Al-Hawas A. Identification of tampering methods among 12,385 Arabian camels using different diagnostic imaging techniques. Open Vet J. (2025) 15:1226–38. doi: 10.5455/OVJ.2025.v15.i3.14, PMID: 40276198 PMC12017717

